# Differentiating cellular leiomyoma from uterine sarcoma and atypical leiomyoma using multi-parametric MRI

**DOI:** 10.3389/fonc.2022.1005191

**Published:** 2022-10-06

**Authors:** Cong Wang, Xianying Zheng, Zuofu Zhou, Yuequan Shi, Qin Wu, Kaiwu Lin

**Affiliations:** Department of Radiology, Fujian Maternity and Child Health Hospital, Fuzhou, Fujian, China

**Keywords:** magnetic resonance imaging, uterine leiomyoma, uterine sarcoma, diffusion-weighted MRI, atypical leiomyoma

## Abstract

**Objectives:**

To evaluate the diagnostic performance of conventional magnetic resonance imaging (cMRI) combined with diffusion-weighted MRI (DWI) in discrimination of cellular leiomyoma, uterine sarcoma, and atypical leiomyoma.

**Methods:**

This retrospective study enrolled 106 patients with uterine masses, including 51 cellular leiomyomas (CLs), 32 uterine sarcomas (USs) and 23 degenerated leiomyomas (LMs) confirmed by histopathologic examination. Clinical data and imaging findings were assessed. Chi-squared test for qualitative variables and one way ANOVA analysis for quantitative variables were performed. Logistic regression analysis and the receiver operating characteristic (ROC) analysis were performed to determine the cut-off point and diagnostic performances for significant numeric values or multiple models.

**Results:**

Morphology (Odds ratio [OR] = 6.36) and margin (OR = 13.84) derived from cMRI were independent indicators for differentiating CLs from USs, and T2WI signal (OR = 0.23) were an independent indicator for differentiating CLs from degenerated LMs (all *P* < 0.05). The cutoff value of apparent diffusion coefficient (ADC) derived from DWI for differentiating CLs from USs was 839 ×10^-6^ mm^2^/sec and was 1239 ×10^-6^ mm^2^/sec for differentiating CLs from degenerated LMs. Compared with the use of cMRI features and ADC value alone, combination of independent indicators and ADC value achieved higher AUCs for both differentiations (all *P* < 0.05).

**Conclusions:**

cMRI is a reliable tool for differentiating CLs from USs and atypical leiomyoma, especially degenerated LMs. The combined use of cMRI and DWI can improve the differential diagnostic performance.

## Introduction

Uterine leiomyomas (LMs) are the most common neoplasms in gynecologic system, occurring in approximately 20%-30% of women of reproductive age and up to 70% of premenopausal women (1, 2). More importantly, up to 65% of LMs are present with varied clinical symptoms and atypical imaging manifestations, including a variety degree of degeneration or cellular histologic subtype (3, 4). Although LMs are typically recognized as benign entities, some atypical LMs, particularly cellular leiomyomas (CLs), have now been defined as borderline tumors with a potential of malignant transformation and a high recurrence rate (5). Therefore, differentiation of CLs from other types of atypical LMs (especially degenerated LMs) and malignant tumors, is of great clinical relevance since their prognosis and therapeutic implications are completely different (1–7). In such condition, uterine sarcomas (USs) which are rare malignant uterine tumors should also be included into clinical differentiation because of their extremely aggressive biology behavior and poor prognosis (8, 9).

Magnetic resonance imaging (MRI) has been recognized as a highly useful modality in the diagnosis, localization, and management determination of this entity (10). Conventional MRI (cMRI) is capable of comprehensively evaluating the localization, morphology, boundary, vascularity, and internal components, especially when paramagnetic contrast is applied (10). Advanced MRI techniques, such as diffusion-weighted MRI (DWI), may supplement conventional imaging with respect to the physiological and functional information obtained (11). As previously reported, DWI holds a potential ability to differentiate uterine sarcomas from benign leiomyomas (11). In a very recent study, Abdel et al. (12) developed an algorithm based on DWI to differentiate benign atypical leiomyomas from malignant uterine sarcomas.

However, to the best of our knowledge, few studies have systematically elucidated the discriminative value of cMRI combined with DWI in distinguishing among atypical LMs, including degenerated LMs and CLs, and USs. Thus, the purpose of this study was to assess the benefit of adding DWI to the conventional MRI for the differential diagnosis of atypical LMs and leiomyosarcomas.

## Materials and methods

### Patients

**Figure 1:**
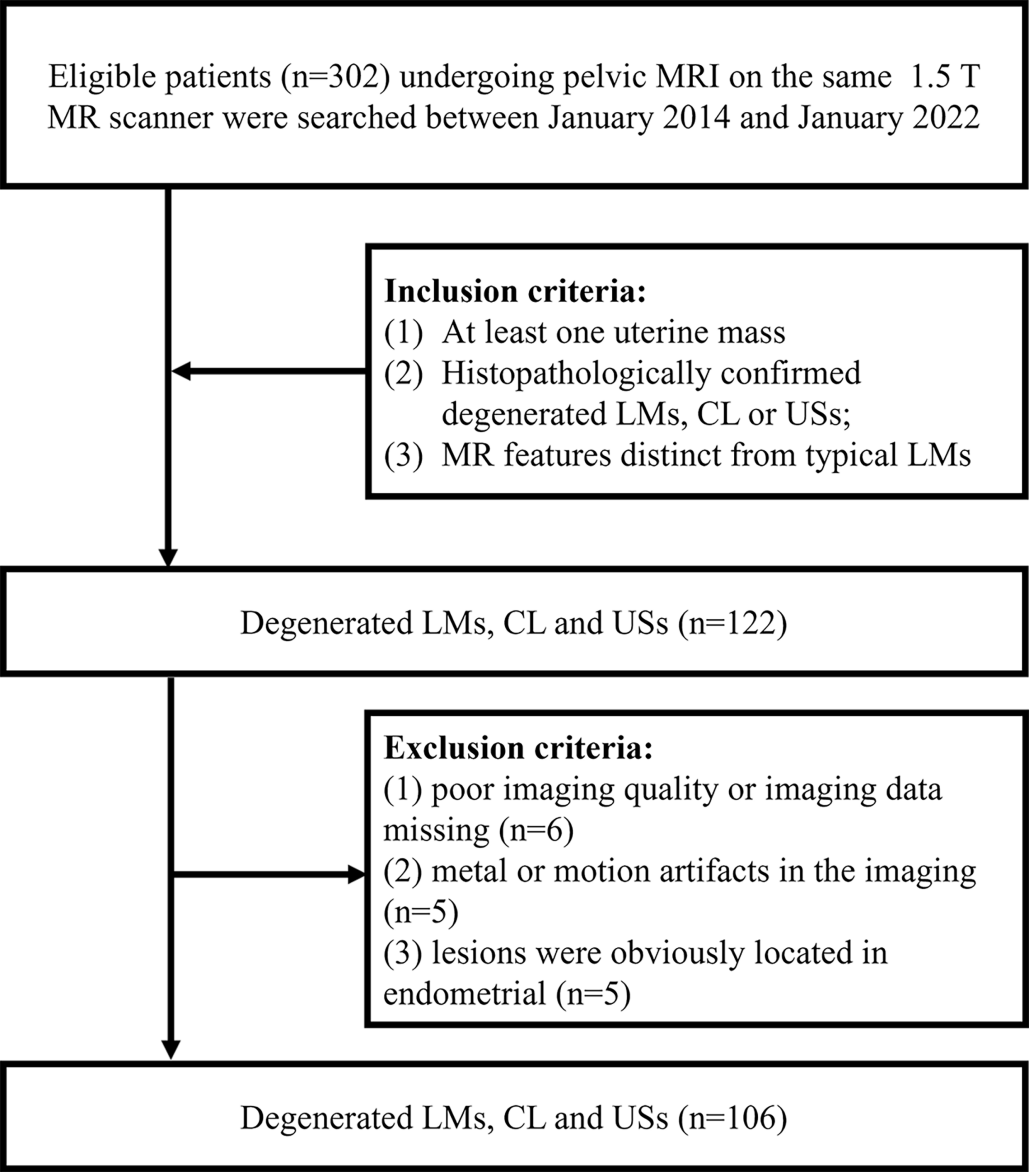
Flowchart showing the patient enrollment process.

This retrospective study was approved by our institutional review board, and the requirements for informed consent forms were waived. We retrospectively reviewed patients who underwent pelvic MRI examination with at least one uterine mass in our center between January 2014 and January 2022. Patients were selected according to the following condition (1): surgically and pathologically proved degenerated LMs, CL or USs (2); MRI features were different from typical leiomyomas. Patients were excluded according to the following condition (1): poor imaging quality or imaging data missing (2); metal or motion artifacts in the imaging (3); lesions were obviously located in endometrial. Finally, 106 patients were enrolled in the study, including 51 CLs, 32 USs and 23 degenerated LMs (**Figure 1**).

### Imaging protocol

MRI examination was performed using a 1.5-T MRI scanner (GE Signa HD MRI system). The conventional nonenhanced MRI protocol consisted of the following sequence: axial gradient-echo T1-weighted sequence (T_1_WI, TR/TE 450 msec/15 msec) with a matrix of 320 × 256; and fat-suppressed T2-weighted (fs-T_2_WI, TR/TE 2800-4200 msec/74-82 msec) sequences in the axial, sagittal and coronal planes with a matrix of 320 × 256; axial DWI (*b* = 800 sec/mm^2^) with a matrix of 128 × 28; axial T1-weighted three-dimensional (3D) gradient-recalled echo (LAVA) multiphase dynamic enhancement sequence (TR/TE 3.5 msec/1.6 msec) was obtained after a rapid intravenous injection of 0.1 mL/kg of gadopentetic acid (0.5 mmol/ml) at an injection rate of 3 mL/s.

### Image analysis

The image assessment was performed by 2 radiologists with more than 10 years of radiographic experience in obstetrics and gynecology. Two radiologists independently evaluated the image manifestations, including (1): the number of the lesion (2); maximum diameter, margin and border (3); hemorrhage, necrosis and degeneration within the lesion; number of the lesion (4); T1WI and T2WI signal intensity of the lesion (5); DWI signal intensity and apparent diffusion coefficient (ADC) value (6); degree of enhancement (7); the thickness of endometrium. The morphology of lesions was described as round/oval and irregular. Maximum diameter measurement was taken in the axial plane. Compared with that of the iliopsoas, T1WI signal intensity, T2WI signal intensity and DWI signal intensity was graded as hypointense, isointense, and hyperintense; T2WI signal intensity was defined as hypointense, isointense, hyperintense. ADC value was assessed in the ADC map by using the circular region of interest (15-25 mm^2^). Avoiding the degeneration, necrotic, and hemorrhage parts within the tumor, several circular regions of interest were placed in the solid area. Then the lowest value of mean ADC in these regions was recorded. Thickened endometrium was defined when the endometrium thickness was more than 10 mm.

### Statistical analysis

All statistical analyses were performed with statistical software (GraphPad Prism, Version 8.1.0). We performed chi-squared test for qualitative variables and one way ANOVA analysis for quantitative variables. The variables that were significantly different among the three groups would be further evaluated with logistic regression analysis. The receiver operating characteristic (ROC) analyses and logistic regression analyses were performed at last to determine the cutoff point, sensitivity, specificity and area under the ROC curves (AUC) for significant numeric values or combined models. Statistical significance was considered when *P* value less than 0.05. Cohen kappa coefficient was used to analyze the interobserver reliability between observer 1 and observer 2: κ< 0.40, poor; 0.40-0.75, fair to good; >0.75, excellent (13).

## Results

**Figure 2:**
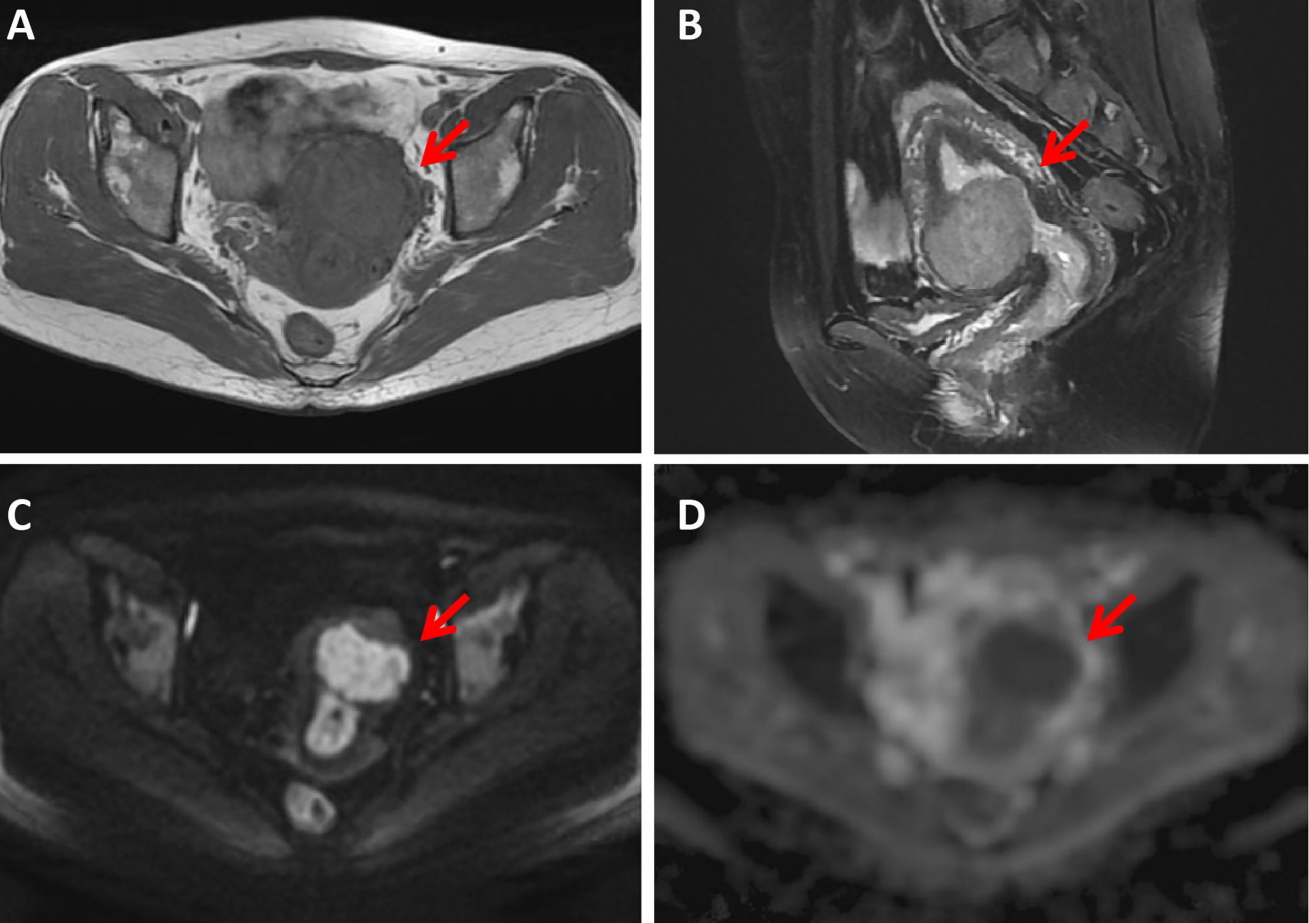
A 43-year-old woman with a cellular leiomyoma. A mass was located in the uterine anterior wall (arrow) with a clear margin, showing isointensity on T1WI **(A)** and hyperintensity on T2WI images **(B)**. This mass showed high signal intensity on diffusion-weighted MR image **(C)** with a low ADC value (ADC = 985 ×10^-6^ mm^2^/sec) **(D)**.

**Figure 3:**
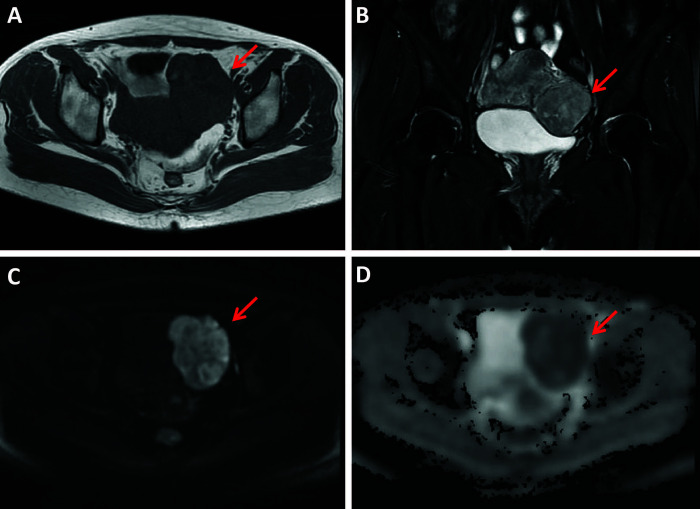
A 49-year-old woman with a uterine sarcoma. A mass was found in the uterine-side wall (arrow) with an unclear margin, showing heterogeneous iso-to-hyperintensity on T1WI **(A)** and heterogeneous hyperintensity on T2WI images **(B)**. The tumor showed high signal intensity on diffusion-weighted MR image **(C)** with a low ADC value (ADC = 736 × 10^-6^ mm^2^/sec) **(D)**.

**Figure 4:**
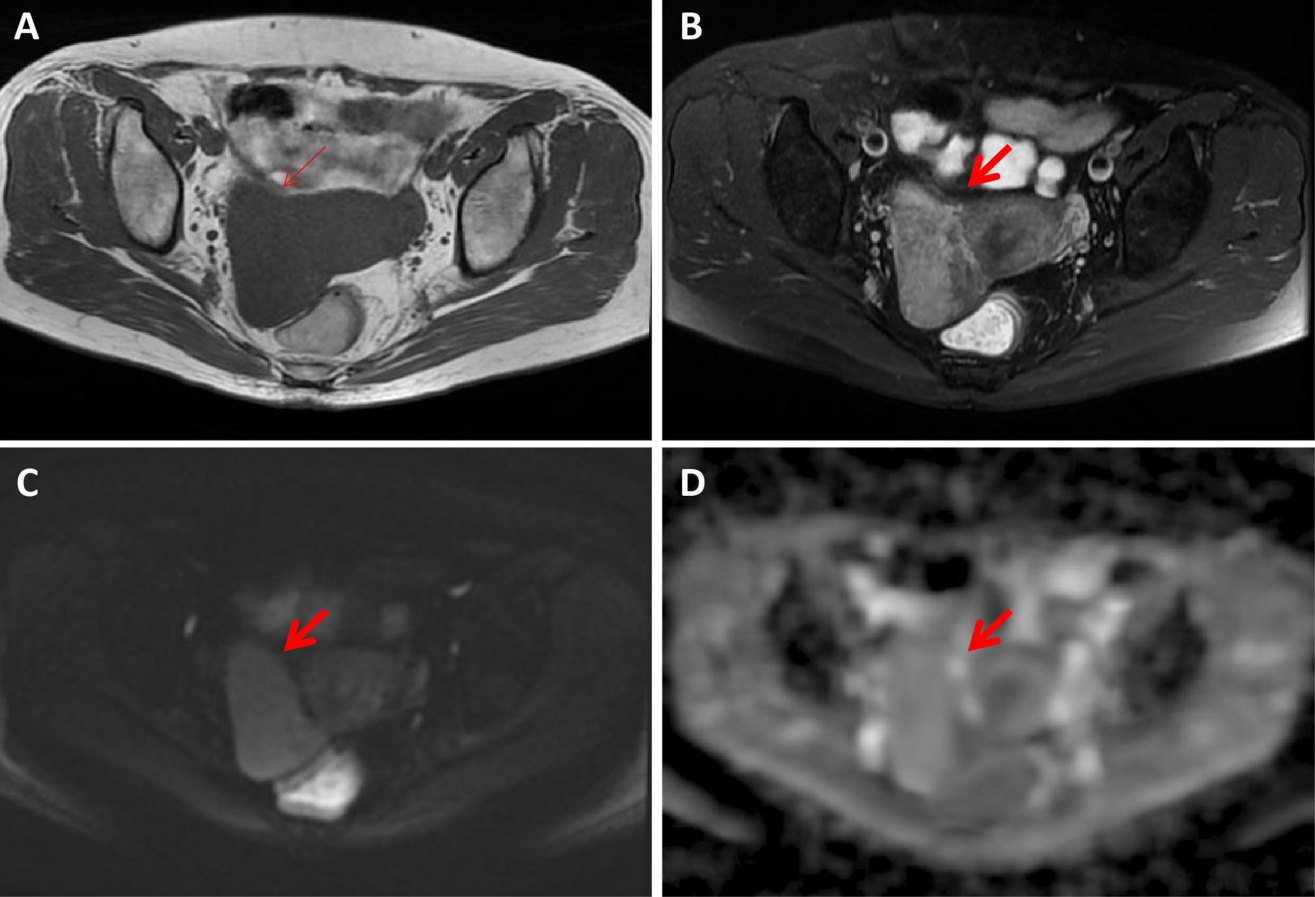
A 56-year-old woman with a hydropic degeneration leiomyoma. The mass was detected in the uterine right wall (arrow) with isointensity on T1WI **(A)** and heterogenous iso-to-hyperintensity on T2WI images **(B)**. The tumor showed high signal intensity on diffusion-weighted MR image **(C)** with a high ADC value (ADC = 1339 × 10^-6^ mm^2^/sec) **(D)**.

The κ values revealed excellent interobserver agreement (all κ > 0.75) for assessing all parameters. The conventional MR and DWI (ADC value) findings of all the lesions are summarized in **Table 1** and representative images are shown in **Figures 2**–**4**. We found significant differences of morphology (*P* < 0.0001) and margin (*P* < 0.0001) among degenerated LMs, CLs and USs, in which LMs tended to display as round/oval and well-defined masses, whereas USs were more likely to be irregular and poorly defined. USs had a predilection for necrosis (18/32, 56.25%) and higher chance of hemorrhage (12/32, 37.5%). There were no significant differences in the probability of multiple lesion occurrences, thickness of endometrial, ascites, and T1WI signal among these three groups (all *P* > 0.05). Additionally, we found no USs were associated with degeneration, and a significant difference between benign and malignant lesions regarding the presence of degeneration (*P* < 0.0001). On T2WI images, the solid portions of both CLs and USs were present as hyperintense, whereas most of degenerated LMs showed hypointensity (*P* < 0.0001). Moreover, compared with degenerated LMs, solid portions of both CLs and USs showed higher DWI signal with lower ADC values (*P* < 0.0001).

**Table 1:** Comparisons of clinical demographics, conventional MRI and DWI/ADC values among CLS, USs and degenerated LMs.

Characteristics	CLs	USs	Degenerated LMs	*P* value
Age	43.0 ± 9.6	48.2 ± 9.2	43.0 ± 9.4	0.046
Number				0.0789
Single	47	25	17	
Multiple	4	7	6	
Morphology				<0.0001
Round/oval	39	5	13	
Irregular	12	27	10	
Margin				<0.0001
Well defined	39	7	15	
Poorly defined	12	25	8	
Endometrial thickness				0.7018
≤1mm	41	28	19	
>1mm	10	4	4	
Hemorrhage				<0.0001
Yes	1	12	4	
No	50	20	19	
Necrosis				<0.0001
Yes	1	18	1	
No	50	14	22	
Degeneration				<0.0001
Yes	11	0	23	
No	40	32	0	
Ascites				0.2881
Yes	4	6	4	
No	47	26	19	
T1WI signal				0.9505
hypointense	1	1	1	
isointense	49	30	21	
hyperintense	1	1	1	
T2WI signal				<0.0001
hypointense	4	3	17	
isointense	2	2	0	
hyperintense	45	27	6	
DWI signal				<0.0001
hypointense	1	0	15	
hyperintense	50	32	8	
ADC value (×10^-6^ mm^2^/sec)	985.8 ± 188.1	838.4 ± 213.5	1451.2 ± 435.1	<0.0001

**Figure 5:**
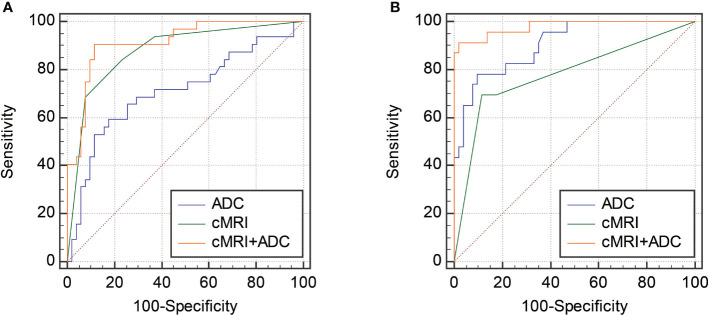
ROC curves showing the diagnostic performances of cMRI, ADC and the combination of cMRI and ADC in differentiating CLs from degenerated LMs **(A)** and USs **(B)**.

As shown in **Table 2**, seven parameters, including patients’ age, morphology, margin, hemorrhage, necrosis, degeneration and T2WI signal, were further enrolled into the multivariate analysis with a forward manner for determining the independent predictors for differentiation of CLs from USs and degenerated LMs. Our multivariate analyses showed that morphology, margin and T2WI signal of the mass were independent predictors of CLs with odds ratios of 6.36, 13.84 and -1.47, respectively (*P* = 0.035, 0.006 and 0.019, respectively). The ROC curve analyses of ADC value for differentiating CLs from USs and degenerated LMs are shown in **Table 3**. The ROC analyses yielded a cutoff ADC value of 839 ×10^-6^ mm^2^/sec, with a sensitivity of 59.38%, a specificity of 82.35% for differentiation of CLs from USs, and a cutoff ADC value of 1239 ×10^-6^ mm^2^/sec, with a sensitivity of 78.26%, a specificity of 90.20 for differentiation of CLs from degenerated LMs (**Figure 5**).

**Table 2:** Logistic regression analysis of clinical demographics and conventional MRI for differentiating CLs from USs and degenerated LMs.

Variables	Coefficient	Odds ratio	95% CI	*P* value
**Differentiation of CLs from USs**				
Morphology	1.85	6.36	1.14-35.67	0.035
Margin	2.63	13.84	2.10-91.26	0.006
**Differentiation of CLs from degenerated LMs**				
T2WI signal	-1.47	0.23	0.067-0.790	0.019

**Table 3:** Measurements of the cut-off value, sensitivity, specificity, and AUC of ADC, conventional MRI parameters, and combination of ADC and cMRI parameters for differentiating CLs from USs and degenerated LMs.

	Cutoff value	Youden index	Sensitivity (%)	Specificity (%)	AUC
**Differentiation of CLs from USs**					
ADC (×10^-6^ mm^2^/sec)	839	0.417	59.38	82.35	0.710
cMRI	–	0.609	68.75	92.16	0.877
cMRI+ADC	–	0.789	90.62	88.24	0.915
**Differentiation of CLs from degenerated LMs**					
ADC (×10^-6^ mm^2^/sec)	1239	0.684	78.26	90.20	0.906
cMRI	–	0.578	69.57	88.24	0.780
cMRI+ADC	–	0.893	91.30	98.04	0.980

To further improve the diagnostic performance, the independent predictors derived from cMRI were combined with ADC for distinguishing CLs from USs and degenerated LMs. As shown in **Table 3** and **Figure 5**, the combination of cMRI parameters (morphology and margin) and ADC value significantly improved the diagnostic performance with an AUC of 0.915, a sensitivity of 90.62% and a specificity of 88.24% (ADC vs. cMRI+ADC: z statistic = 3.305, *P* < 0.0001; cMRI vs. cMRI+ADC: z statistic = 2.292, *P* = 0.022) for the differentiation of CLs from USs. The combination of T2WI signal from cMRI and ADC value also significantly improved the diagnostic performance for the discrimination of CLs from degenerated LMs with an AUC of 0.980, a sensitivity of 91.30% and a specificity of 98.04% (ADC vs. cMRI+ADC: z statistic = 2.083, *P* = 0.037; cMRI vs. cMRI+ADC: z statistic = 3.820, *P* < 0.0001).

## Discussion

The incidence of CLs is low, and their signs and symptoms are non-specific, whereas the biological behavior of CLs is borderline, showing a potential of malignant transformation and a high recurrence rate (14). Hence, differentiation of CLs from other atypical CLs and USs is crucial for selecting optimal treatment strategies and improving prognosis of patients. In this current study, we systematically investigate the characteristics from cMRI and found that irregular morphology, ill-defined margin, and hyperintense signals on T2WI were most valuable features that could dramatically differentiate CLs from USs or degenerated LMs. With the combination of cMRI characteristics and ADC value derived from DWI, optimal sensitivity and specificity can be achieved in distinguishing these entities.

Uterine LMs are histologically composed of smooth muscle cells with little or no mitotic activity; on the other hand, CLs were defined as an atypical subset of uterine leiomyomas with higher cellularity than the adjacent myometrium. In clinic, diagnosis of typical LMs is not difficult when lesions in uterine have imaging characteristics, such as isointense T1 signal with regular morphology and well-defined margins (15). However, when LMs present atypical imaging manifestations, particularly with degeneration, the accurate differentiation will be very challenging (16, 17). In this study, we found both CLs and degenerated LMs can be associated with degeneration or cystic changes, inducing hyperintensity on T2WI images. However, for the solid portion of the uterine masses, we found CLs were more likely to show a global or focal hyperintensity on fs-T2WI images compared with degenerated LMs. Recent reviews have shown a significantly higher signal on T2-weighted images in hypercellular uterine tumors in comparison to benign leiomyomas, which generally demonstrate homogenously low signal on T2-weighted images (18, 19), which further helps in explaining in the findings present in this study, as CLs are increasingly recognized as a borderline tumor with hypercellularity. Additionally, CLs is composed of densely cellular fascicles of smooth muscle with little intervening collagen (20). Mitotic figures are few, and there is little or no cytologic atypia (20). Its hypercellular nature with little collagenous tissue may both contribute to signal increase on T2WI images. Moreover, an ADC value of 1239 ×10^-6^ mm^2^/sec or less might indicate the diagnosis of CLs without manifestation of malignant tumor, which was consistent with a previous study (20). In that study, Takeuchi et al. (20) reported that the ADC value of CLs were significantly lower than that of degenerated LMs. Our finding indicated that the biological components of CLs are different from those of degenerated LMs. LMs with degeneration can still be considered as benign LMs which enriched in extracellular matrix with abundant collagen types I-III, whereas CLs are more hypercellular, resulting in higher prevalence of high signal intensity on T2WI images and lower ADC values (14). Of note, even though ADC can provider a slightly higher sensitivity and specificity, the diagnostic performance of ADC and cMRI is comparable. However, when we combined cMRI with ADC value from the solid portion of the mass, the diagnostic performance can be significantly improved with an AUC of 0.980.

Moreover, a recent case-control study showed that CLs had a distinct clinical phenotype from LMs and showed some characteristics shared with USs (5). Thus, CL may be recognized as a subgroup of leiomyoma variants where benign disease evolves to malignancy (5). Importantly, in this present study, we found both cMRI features, including morphology and margin, are independent indicators of USs. Specifically, when the uterine mass is associated irregular morphology and ill-defined margin, it highly suggests a possibility of US, which were in good line with previous studies (14, 16). Histopathologically, USs are aggressive malignant tumors which can easily invade the normal surrounding tissue, demonstrating the irregular and ill-defined margins. Additionally, compared with CLs, USs had a lower ADC value with a cutoff of 839 ×10^-6^ mm^2^/sec. It was not surprising that CLs would be associated with higher ADC values compared with USs due to lower cellularity. However, promisingly, when ADC was added into the diagnostic flow of cMRI, the diagnostic performance of differentiating CLs from USs can be significantly improved with an AUC of 0.915.

Our study has several limitations. First, the number of patients with sarcomas was relatively small due to its extreme rarity. Second, this retrospective study was conducted without validation. Selection bias should be taken into consideration. An external and/or prospective validation with more numerous patients will be performed to translate our results into the clinic. Third, considering the rarity of uterine CLs, disease prevalence and a grade of suspicion would have influence on the results of the MRI systems.

In conclusion, we presented characteristic MRI features of among cellular leiomyomas, degenerated leiomyomas and uterine sarcomas. We also proved the ADC value of these lesions could assist the diagnosis and differentiate cellular leiomyoma from uterine sarcoma and atypical leiomyoma. The combination of cMRI and ADC value can be a reliable tool for distinguishing these entities, which is useful for optimization of treatment strategies for uterine tumor, avoiding inappropriate less invasive treatment options.

## Data availability statement

The original contributions presented in the study are included in the article/Supplementary Material. Further inquiries can be directed to the corresponding author.

## Ethics statement

The studies involving human participants were reviewed and approved by the ethical committee of Fujian Maternity and Child Health Hospital. Written informed consent for participation was not required for this study in accordance with the national legislation and the institutional requirements.

## Author contributions

Study concept and design: CW and KL. Acquisition of data: CW, QW, and KL. Resources: CW, XZ, ZZ, YS, and KL. Analysis and interpretation of data: CW and KL. Drafting of the manuscript: CW. Critical revision of the manuscript for important intellectual content: CW and KL. Technical or material support: CW, XZ, ZZ, YS, and KL. Study supervision: KL. All authors read and approved the final manuscript.

## Acknowledgments

The authors thank Dr. Zebin Xiao (MD, PhD), a principal investigator and professor at the University of Pennsylvania for kindly proofreading.

## Conflict of interest

The authors declare that the research was conducted in the absence of any commercial or financial relationships that could be construed as a potential conflict of interest.

## Publisher’s note

All claims expressed in this article are solely those of the authors and do not necessarily represent those of their affiliated organizations, or those of the publisher, the editors and the reviewers. Any product that may be evaluated in this article, or claim that may be made by its manufacturer, is not guaranteed or endorsed by the publisher.
